# Environmental Exposure to Dioxins, Dibenzofurans, Bisphenol A, and Phthalates in Children with and without Autism Spectrum Disorder Living near the Gulf of Mexico

**DOI:** 10.3390/ijerph14111425

**Published:** 2017-11-21

**Authors:** Mohammad H. Rahbar, Hanes M. Swingle, MacKinsey A. Christian, Manouchehr Hessabi, MinJae Lee, Meagan R. Pitcher, Sean Campbell, Amy Mitchell, Ryan Krone, Katherine A. Loveland, Donald G. Patterson

**Affiliations:** 1 Department of Epidemiology, Human Genetics and Environmental Sciences (EHGES), University of Texas School of Public Health at Houston, Houston, TX 77030, USA; 2 Division of Clinical and Translational Sciences, Department of Internal Medicine, University of Texas McGovern Medical School at Houston, Houston, TX 77030, USA; MinJae.Lee@uth.tmc.edu; 3 Biostatistics/Epidemiology/Research Design (BERD) Component, Center for Clinical and Translational Sciences (CCTS), University of Texas Health Science Center at Houston, Houston, TX 77030, USA; Mackinsey.A.Christian@uth.tmc.edu (M.A.C.); Manouchehr.Hessabi@uth.tmc.edu (M.H.); 4 Division of Developmental-Behavioral Pediatrics, University of South Alabama, Mobile, AL 36604, USA; hswingle@health.southalabama.edu (H.M.S.); abmitchell@health.southalabama.edu (A.M.); rkrone@health.southalabama.edu (R.K.); 5 Baylor Licensing Group, Baylor College of Medicine, Houston, TX 77030, USA; Meagan.Pitcher@bcm.edu; 6 AXYS Analytical Services Ltd., Sidney, BC V8L5X2, Canada; scampbell@axys.com (S.C.); dpatterson@axys.com (D.G.P.); 7 Department of Psychiatry and Behavioral Sciences, University of Texas McGovern Medical School at Houston, Houston, TX 77054, USA; Katherine.A.Loveland@uth.tmc.edu

**Keywords:** autism spectrum disorder (ASD), dioxins, dibenzofurans, bisphenol A, phthalates, neurodevelopmental disorder, Gulf of Mexico, children

## Abstract

Environmental exposure to organic endocrine disrupting chemicals, including dioxins, dibenzofurans, bisphenol A (BPA), and phthalates has been associated with neurodevelopmental disorders, including autism spectrum disorder (ASD). We conducted a pilot monitoring study of 30 ASD cases and 10 typically developing (TD) controls ages 2–8 years from communities along the Gulf of Mexico near Alabama, which houses 14 Superfund sites, to assess the concentrations of dioxins and dibenzofurans in serum, and BPA and phthalate ester metabolites in urine. Based on General Linear Models, the lipid- or creatinine-adjusted geometric mean concentrations of the aforementioned chemicals did not differ between the ASD case and TD control groups (all *p* ≥ 0.27). We compared our findings to the adjusted means as reported by the National Health and Nutrition Examination Survey, survey years 2011–2012, and found that TD controls in our study had lower BPA (59%) and MEHHP (26%) concentrations, higher MBP (50%) concentration, and comparable (<20% difference) MEP, MBZP, MEOHP, and MCPP concentrations. We also conducted a preliminary investigation of dietary exposures and found that the consumption of certain types of fish may be associated with higher OCDD concentrations, and the consumption of soft drinks and juices may be associated with lower BPA and MEOHP concentrations, respectively.

## 1. Introduction

Environmental exposure to organic endocrine disrupting chemicals (EDCs) [1] and persistent organic pollutants [2], including dioxins [3], dibenzofurans [4], bisphenol A (BPA) [5,6], and phthalates [7,8], has been associated with neurodevelopmental disorders [9,10,11]. In previous studies, perinatal exposure to dioxins and early postnatal exposure to dibenzofurans as measured in maternal breast milk were associated with poor neurodevelopmental outcomes, including impaired motor coordination and cognitive ability [3] and autistic traits [12], in children. Children with neurodevelopmental disorders also had higher concentrations of phthalates in serum [11], and higher concentrations of BPA in serum [11], urine [13], and plasma [14], though these exposures were measured after diagnosis and cannot be used to draw conclusions on the contribution of phthalates and BPA to etiology of neurodevelopmental disorders.

Autism spectrum disorder (ASD) is a neurodevelopmental disorder whose etiology may be associated with the exposure to EDCs [3,8,10,15], either alone or through gene-environment interactions [16] and epigenetic changes [17]. Epidemiologic studies have reported associations between urinary phthalates and phthalate metabolites with increased levels of lipid peroxidation, inflammation, and decreased levels of antioxidants [18,19], which are markers of oxidative stress. Since oxidative stress is suspected to play a role in ASD etiology [20], these findings raise questions as to if through a similar mechanism, exposure to dioxins, dibenzofurans, BPA, and phthalates is associated with neurodevelopmental disorders, such as ASD.

The level of exposure to dioxins and dibenzofurans in communities along the coast of Alabama is of concern for several reasons. Between 2009 and 2012 Alabama houses 14 Superfund sites, 45–46 facilities that released dioxin and dioxin-like compounds, and three facilities that released dibenzofurans into the environment, contributing to an 81% increase of dioxins and dioxin-like compounds in Alabama by 2011 [21]. Additionally, Alabama borders the Florida panhandle and its paper mills, which has dioxin and dibenzofuran sediment levels of up to 77.51 ppt [22]. As a result, dioxin concentrations in sediment and subsequent bioaccumulation in the food chain have been a concern for several years [23]. Furthermore, the BP Deepwater Horizon Oil Spill (DWHOS) in 2010 released roughly 3.19 million barrels of oil into the northern Gulf of Mexico [24]. The deliberate oil burns from clean-up efforts created polychlorinated dioxins and dibenzofurans that were distributed to the atmosphere in smoke flumes, potentially affecting air quality [25,26]. The oil spill and cleanup efforts also raised concern about seafood contamination and subsequent risks to health in nearby communities along the Gulf of Mexico [27,28], particularly since the major route of human exposure to dioxins is via bioaccumulation through the food chain [29], including meats and fish [4]. The potential health impacts of the DWHOS are of such concern that the National Institute of Environmental Health Sciences (NIEHS) funded a multi-site consortium, the Gulf Coast Health Alliance: Health Risks related to the Macondo Spill (GC-HARMS), to measure the distribution of hazardous chemicals that remain along the coast by sampling fish and shellfish in the Gulf of Mexico [30]. 

Chlorinated Dibenzo-p-dioxins (CDDs) [31] are classified into eight groups, according to the number of chlorine atoms [32]. The CDDs that were analyzed in this study belonged to the tetra-chlorinated dioxin (TCDD), pentachlorinated dioxin (PECDD), hexa-chlorinated dioxin (HXCDD), hepta-chlorinated dioxin (HPCDD), and octa-chlorinated dioxin (OCDD) groups. Dioxins and structurally related dioxin-like chemicals, including polychlorinated dibenzofurans (PCDFs) [33], are persistent environmental chemicals that are the byproduct of industrial processes, including incineration, chlorine bleaching of paper and pulp, manufacture or disposal of chlorine-containing products, and the manufacture of some pesticides, herbicides, and fungicides, and can also result from natural processes, including forest fires [33,34]. 

BPA and phthalates are endocrine disrupting chemicals that can impair neurologic processes despite their short half-lives, ranging from hours to days for phthalates and less than one day for BPA [35]. These chemicals are widely used in the production of consumer plastic products, including food and water packaging [36]. In addition to plastic products, humans may be exposed to phthalates through household dust [37], water, and food [38]. A major dietary route of exposure for children is through fish, meat, and milk [39]. 

We conducted a pilot study to assess the feasibility of conducting a large scale population-based study of communities along the Alabama coast of the Gulf of Mexico to investigate the role of environmental chemical contaminants, such as dioxins, dibenzofurans, BPA, and phthalates, and their interactions with genes that are involved in contaminant metabolism in relation to ASD. This pilot project revealed difficulty in enrolling suitable typically developing (TD) children as controls, and as a result, we have 30 ASD cases and 10 TD controls in the present study. 

The descriptive data provided here will lay the foundation for the first comprehensive epidemiological study of the association between the exposure to dioxins, dibenzofurans, BPA, and phthalates and ASD in children living near the Gulf of Mexico. In the present manuscript, we provide hypothesis-generating data on the concentrations of these contaminants in serum or urine samples collected from children with and without ASD in communities in this region, and compare these concentrations to national averages as reported by the National Health and Nutrition Examination Survey (NHANES). Since the population along the Gulf of Mexico is expected to be at a higher risk of exposure to EDCs through several sources, particularly through food consumption, we also report preliminary results comparing the mean concentrations of these chemicals in children with various dietary exposures. 

## 2. Materials and Methods

### 2.1. General Description

This pilot study was designed as a 1:1 age-, race-, and sex-matched case-control study of children with ASD and TD controls aged 2–8 years. From July 2015–Septemeber 2016, we enrolled 30 ASD cases and 10 TD controls. To be eligible for the present study, all of the children had to have been born along the Gulf of Mexico in or near Alabama (e.g., Alabama, Florida, or Mississippi). From July 2015–September 2016, parents or guardians of children, ages 2–8 years inclusive, who had previously been diagnosed with ASD based on the Autism Diagnostic Observation Schedule (ADOS) [40], DSM-5, and clinical expert judgment at the University of South Alabama (USA) Autism Diagnostic Clinic were asked to participate during routine clinic follow-up visits. To confirm that the clinical diagnosis met DSM IV or DSM V criteria at the time of enrollment, we administered both the Autism Diagnostic Interview-Revised (ADI-R) [41] and Autism Diagnostic Observation Schedule-2 (ADOS-2) [42]. For cases who had been evaluated using ADOS prior to the release of the revised second edition (ADOS-2), the original ADOS score was recalculated using the ADOS-2 scoring algorithms. TD control children were recruited from the USA General Pediatric clinics and through advertisements on social media (e.g., Facebook) and in a local newspaper, and were matched to ASD cases based on age (±6 months), race, and sex. The Lifetime and Current forms of the Social Communication Questionnaire (SCQ) [43] were administered to the parents/guardians of TD control children to rule out symptoms of ASD using a cut-off point of 6, which is one standard deviation below the mean SCQ score of TD school children [44]. Parental/guardian consent was obtained for all of the children. Children ages 7–8 also provided written assent when it was deemed appropriate based on the child’s cognitive level. 

A questionnaire was administered to the parents/guardians of both ASD cases and TD controls to collect demographic and socioeconomic status (SES), including information about parental age and education levels. In addition, food frequency data representing the typical consumption of food items by children were collected. This included vegetables, fruits, meat, and fresh and canned seafood, with a particular focus on potential exposure to dioxins, dibenzofurans, BPA, and phthalates. In addition, we collected 2 mL of urine for BPA and phthalate ester metabolite (PEM) analysis, 1 mL of urine for assessment of creatinine (used to adjust BPA and PEM concentrations by creatinine concentrations), and 15–20 mL of whole blood to obtain 7–10 mL of serum for assessment of dioxins and dibenzofurans from all of the children. Each family was given a $10 gift card for their participation.

For assessment of creatinine in urine, the urine samples were analyzed at LabCorp Laboratory in Mobile, Alabama immediately after collection. The urine samples for BPA and phthalates were frozen and stored at −10 °C and serum samples for dioxins and dibenzofurans were frozen and stored at −30 °C until they were transported to SGS AXYS Analytical Services Ltd., in Sidney, BC, Canada. The samples were analyzed for dioxins and dibenzofurans, BPA, and phthalate concentrations using standard methodology as approved by the Canadian Association for Laboratory Accreditation (CALA) [45] and the National Environmental Laboratory Accreditation Program (NELAP) [46], and adapted by SGS AXYS Analytical Services Ltd. Institutional Review Boards (IRBs) of the University of Texas Health Science Center at Houston (Project identification code: HSC-GEN-14-0792) and USA (Project identification code: 14-344) approved this study. The data presented herein represent analysis of 30 ASD cases and 10 TD control children. 

### 2.2. Assessment of Polychlorinated Dibenzodioxins and Dibenzofurans (Dx/F)

Prior to analysis a 300 µL sub-sample was removed and submitted to Quebec Toxicology for total lipid analysis. All of the remaining sera from each participant was analyzed by high resolution gas chromatography with high resolution mass spectrometric detection (HRGC/HRMS) (Micromass Autospec Premier, Waters Corporation, Milford, MA, USA). Quantification of target analytes was performed by isotope dilution. Results were reported on a mass of analyte per mass of sera basis. Lipid data from each participant was used to adjust sample size from mass of sera to mass of lipid and the results were reported on a mass of analyte per mass of lipid basis.

### 2.3. Assessment of Bisphenol A (BPA) and Phthalate Ester Metabolites (PEMs)

One milliliter of urine from each participant was extracted and analyzed on a high performance liquid chromatograph coupled to a triple quadrupole mass spectrometer LC-MS/MS (Micromass Quattro Ultima, Waters Corporation, Milford, MA, USA). Quantification was performed by the internal standard/isotope dilution quantification method. Results were reported on a mass of analyte per volume of urine.

### 2.4. Statistical Analysis

We conducted descriptive analyses to examine the distributions of various characteristics of the study population, including demographic characteristics and socioeconomic status (SES), and compared the distribution of these characteristics between ASD cases and TD controls using logistic regression. Children with missing data were excluded from analyses. Due to the limited sample size, we did not adjust for potential confounders in the following analyses in order to avoid unstable estimates. Additionally, we did not account for the potential correlation between paired observations since less than half of the study sample was paired.

A sample-specific Limit of Detection (LoD) was reported by SGS AXYS for each serum and urine sample when analyzed for dioxins/dibenzofurans and BPA/phthalates, respectively. Therefore, the LoDs for each analyte are herein reported as a range of the sample-specific LoDs. If the overall percentage of samples with concentrations below their sample-specific LoD was less than 20%, then we replaced those non-detectable sample concentrations with the sample-specific LoD divided by the square root of two [47]. This is the same method for imputing observations below LoDs used by NHANES [48].

Since the distributions of serum concentrations of dioxins and dibenzofurans and urine concentrations of BPA and PEMs were skewed, we transformed the data using the natural logarithm (ln) in order to produce a distribution that better approximated a normal distribution. The means that the ln transformed concentrations were transformed to their original scale (i.e., pg/g for dioxins and dibenzofurans and ng/mL for BPA and PEMs) by applying an exponential function, called the geometric mean (i.e., exp[Mean (ln OCDD)] = geometric mean OCDD). All mean analyte concentrations reported herein are in reference to the geometric mean. Additionally, the serum dioxin and dibenzofuran concentrations were adjusted per gram of lipid to account for the lipophilic properties of dioxins and dibenzofurans [49]. Similarly, the urine BPA and PEM concentrations were adjusted per gram of creatinine to account for the variation in dilution of urine samples [50]. General Linear Models (GLMs) were used to test the difference between geometric mean concentrations of dioxins, dibenzofurans, BPA, and PEMs between ASD cases and TD controls. We also compared the lipid- or creatinine-adjusted geometric mean concentrations in our study to those reported by NHANES for children of the most similar age group and the most recent survey year that data are available for each group of chemicals. Furthermore, we used GLM to examine the difference in geometric mean concentration of select analytes between those who had dietary exposures, such as consuming seafood, meat, dairy products, and canned food, and those who did not have these dietary exposures. All statistical testing were conducted at 5% level of significance. All of the statistical analyses were conducted using SAS 9.4 [51].

## 3. Results

Among children with ASD and TD children, 80% were male. The mean age of children with ASD was 78.7 months, and the mean age of TD children was 70.5 months. More than half of children with ASD (56.7%) and TD children (70.0%) were Caucasian, while the remaining children were African American. We also found that 50% of parents of children with ASD reported home ownership as compared to 70% home ownership among parents of TD children. However, none of the demographic characteristics of children and their parents reported in Table 1 were significantly associated with ASD status.

Of the seven dioxin congeners that were analyzed in this study, only octa-chlorinated dioxin (OCDD) was detected in 100% of the serum samples, with lipid-adjusted mean (SD) concentrations of 78.51 (2.26) pg/g for children with ASD and 90.93 (1.50) pg/g for TD children (*p* = 0.59). None of the remaining dioxins or dibenzofurans that were analyzed in this study were detectable in at least 70% of serum samples. We compare our results to those most recently reported by NHANES (survey years 2003–2004) for the most similar age group (12–19 years) [48]. The lipid-adjusted LoD ranges for dioxins and dibenzofurans in this study and NHANES lipid-adjusted means and LoDs are displayed in Table 2.

BPA was detected in 81.8% of urine samples, with creatinine-adjusted mean (SD) concentrations of 1.33 (2.10) µg/g for children with ASD and 0.93 (2.43) µg/g for TD children. Of the 10 PEMs analyzed, two were detected in 100% of urine samples: MBP with creatinine-adjusted mean (SD) concentrations of 41.82 (1.88) µg/g in children with ASD and 41.49 (2.60) µg/g in TD children; and, MEOHP with creatinine-adjusted mean (SD) concentrations of 9.76 (2.52) µg/g in children with ASD and 8.23 (2.13) µg/g in TD children. Additionally, four PEMs were detected in at least 70% of urine samples: MEP with creatinine-adjusted mean (SD) concentrations of 28.93 (1.86) µg/g in children with ASD and 37.61 (7.58) µg/g in TD children; MBzP with creatinine-adjusted mean (SD) concentrations of 19.01 (2.38) µg/g in children with ASD and 12.55 (3.07) µg/g in TD children; MEHHP with creatinine-adjusted mean (SD) concentrations of 14.94 (2.45) µg/g in children with ASD and 11.08 (3.22) µg/g in TD children; and, MCPP with creatinine-adjusted mean (SD) concentrations of 4.04 (1.74) µg/g in children with ASD and 4.49 (1.75) µg/g in TD children. We compare our results to those most recently reported by NHANES (survey years 2011–2012) for the most similar age group (6–11 years). The LoD ranges and detectable mean concentrations for BPA and PEMs in this study and the NHANES creatinine-adjusted means and LoDs are reported in Table 3.

A comparison of lipid-adjusted mean OCDD concentrations between those with different dietary exposures revealed the following findings. The lipid-adjusted mean OCDD concentration of children who consumed canned sardine or mackerel was significantly higher than that of children who did not consume canned sardine or mackerel (263.75 pg/g vs. 78.24 pg/g, *p* = 0.02). Similarly, our data showed that lipid-adjusted mean OCDD concentration of children who ate shellfish (lobster, crab, crawfish) was significantly higher when compared to that of children who did not eat shellfish (124.58 pg/g vs. 69.62 pg/g, *p* = 0.02). Analysis also showed that children who drank milk had significantly lower lipid-adjusted mean OCDD concentrations as compared to children who did not drink milk (76.07 pg/g vs. 183.76 pg/g, *p* = 0.01). There were no significant differences in lipid-adjusted mean OCDD concentrations for children who did and did not consume the remaining food items, as displayed in Table 4 (all *p* ≥ 0.09).

Creatinine-adjusted mean BPA concentrations in urine were compared between children that did and did not consume various foods or drink products, such as bottled water, canned fish, bottled juices or soft drinks, and other canned food products. We found that children who drank soft drinks had lower creatinine-adjusted mean BPA concentrations when compared to children who did not drink soft drinks (0.82 µg/g vs. 1.96 µg/g, *p* < 0.01). Our data show that there is no significant difference between the creatinine-adjusted mean BPA concentrations for those who did and did not have other dietary exposures (all *p* ≥ 0.10). Comparisons of creatinine-adjusted mean BPA concentrations for dietary exposure variables are displayed in Table 5.

Similarly, we compared creatinine-adjusted mean MEOHP concentrations in urine between those who did and did not consume bottled water, canned fish, bottled juices/soft drinks, and other canned foods. Children who drank juices (e.g., orange, tomato, etc.) had lower creatinine-adjusted mean MEOHP concentrations as compared to children who did not drink juices (8.31 µg/g vs. 17.79 µg/g, *p* = 0.05). However, our data showed that there is no significant difference between the creatinine-adjusted mean MEOHP concentrations of those who did and did not have other dietary exposures, as listed in Table 6 (all *p* ≥ 0.15). 

## 4. Discussion

Although the sample size was limited in this pilot study, we have reported concentrations of 7 dioxin congeners and 10 dibenzofurans in serum, and BPA and 10 phthalate ester metabolite concentrations in urine of children with and without ASD, ages 2–8 along the Gulf of Mexico as a reference for future studies. However, these results were not adjusted for potential confounders and therefore should be interpreted as exploratory. Additionally, it is important to keep in mind that since we assessed exposure to these chemicals after ASD diagnosis, we cannot make any comments on the contribution of these chemicals to the etiology of ASD, as we only investigated possible associations. We also compared these concentrations to the lipid- or creatinine-adjusted geometric mean concentrations reported by NHANES for children of the most similar age group. In addition, we explored the relationship between dietary exposures and these chemical concentrations. In the following, we will discuss our findings that are related to each of these topics separately for each group of chemicals.

### 4.1. Dioxin and Dibenzofuran Concentrations in Serum of Children Ages 2–8 Living near the Gulf of Mexico

Our results showed that the only dioxin with 100% of samples above LoD (range = 8.41–31.0 pg/g) was OCDD, with lipid-adjusted mean concentrations of 78.51 pg/g of lipid for children with ASD and 90.93 pg/g of lipid for TD children, however this difference was not statistically significant. NHANES, which is a population based study in the US, most recently reported that the lipid-adjusted mean concentrations of OCDD in the 12–19 year age group were unable to be calculated due to a large proportion of results being below LoD (218.0 pg/g) in the 2003–2004 survey years [48]. However, the LoD that was reported in NHANES 2003–2004 survey years was much higher than the sample-specific LoDs that was reported in our study (218 pg/g vs. 8.41–31.0 pg/g). Therefore, we are unable to conclude if the lipid-adjusted mean OCDD concentrations in children 2–8 years living near Gulf of Mexico is similar to that of those ages 12–19 years as reported by NHANES from 2003 to 2004. Based on Toxic Equivalency Factors (TEFs), which are used globally to measure toxicities of dioxins [52,53], OCDD has low toxicity, with a TEF of 0.0003 [54]. OCDD has been found to have adverse effects on development in animals [32], however no human deaths have been attributed to exposure to OCDD or any other dioxins [55]. In comparison, 2,3,7,8-TCDD is the most toxic [55], with a TEF of 1 [53]. Of all the dioxins and dibenzofurans that are included in this study that were also reported by NHANES for the 12–19 year age group in survey years 2003–2004, 1,2,3,4,6,7,8-HpCDD was the only dioxin and 1,2,3,4,6,7,8-HpCDF was the only dibenzofuran with detectable mean concentrations (16.7 pg/g and 9.36 pg/g, respectively) [48]. None of the dibenzofurans analyzed in our study were detectable in at least 70% of serum samples. 

### 4.2. Routes of Exposure for Chlorinated Dibenzo-p-Dioxins (CDDs)

Although there may be some natural sources of dioxins in the environment, such as forest fires, a majority can be contributed to human activity, including the combustion of fossil fuels, industrial waste, bleaching used in paper production, and most importantly, incineration [55]. Dioxins are present globally in soil, water, sediment, plants, and meat. Dioxins in surface waters and sediments can lead to bioaccumulation in the aquatic food chain. However, bioaccumulation factors tend to decrease with dioxins having more than four chlorines [55]. A study conducted in Russia found that a local chemical plant was a source of dioxins detected in air, soil, water, vegetables, and milk, with the most common dioxin being OCDD [56]. Dietary routes of exposure for humans include the consumption of dairy products, meat, pork fat, and fish [56]. In our study, we found detectable concentrations of OCDD in 100% of samples, and those who ate shellfish or canned sardine and mackerel had higher lipid-adjusted mean OCDD concentration when compared to those without these dietary exposures. In contrast with what would be expected, those who drank milk had lower lipid-adjusted mean OCDD concentration when compared to those who did not drink milk, although this result may be skewed because one of the few children who did not drink milk had a very high lipid-adjusted OCDD concentration. This extreme observation was verified to be true. 

### 4.3. BPA and Phthalate Ester Metabolite Concentrations in Urine of Children Ages 2–8 along the Gulf of Mexico

Although not statistically significant, in this study the creatinine-adjusted mean BPA concentration was 43% greater in children with ASD when compared to TD controls (1.33 µg/g vs. 0.93 µg/g). Similarly, in a study in New Jersey, USA, Stein et al. reported significantly higher mean BPA urine concentrations in ASD cases as compared to controls after controlling for gender, age, body mass index (BMI), and creatinine levels as potential confounders [13]. Kardas et al. also reported significantly higher mean BPA concentrations among ASD cases compared to controls in Turkey [11]. However, results from these studies are not directly comparable to ours due to the different methods that are used for adjusting for creatinine [13] and the measurement of BPA in serum [11], respectively. The mean BPA concentrations in the present study are lower for both ASD cases (41%) and TD controls (59%) than the creatinine-adjusted mean BPA concentration for the 6–11 year age group reported by NHANES survey years 2011–2012 (2.27 µg/g). However, these results are consistent with the decreasing trend in geometric BPA concentrations from NHANES survey years 2003–2004 to 2011–2012 [48]. In 2013, NHANES conducted a pilot study to assess the feasibility of assessing the concentration of BPA and PEMs among American children aged 3–5 years, which is a more comparable age range to the children included in the present study. This NHANES pilot study reported a higher creatinine-adjusted mean BPA concentration (3.0 µg/g) among children 3–5 years than we reported in our study, however the NHANES pilot study included a convenience sample of *n* = 122 children and was not population-based [57]. 

Creatinine-adjusted geometric mean concentration for MBzP for children with ASD (19.01 µg/g) was about 50% higher than the concentration for MBzP for TD children (12.55 µg/g), although this difference was not statistically significant. Findings from NHANES (2011–2012) also include the adjusted geometric mean MBzP concentration of 12.5 µg/g for children age 6–11 years in the US, which is comparable to the TD group and is 52% lower than the ASD group from our study [48]. The NHANES pilot study on children 3–5 years reported an adjusted geometric mean MBzP concentration of 13.7 µg/g, which is also comparable to our TD group and lower than the ASD group [57]. In our study, we reported the adjusted geometric mean concentration for MBP, which is the sum of MiBP and MnBP, for ASD cases and TD children (41.82 and 41.49 µg/g, respectively). NHANES did not publish data on MBP concentrations, however they reported creatinine-adjusted geometric mean concentrations for MiBP and MnBP separately for the 6–11 year age group in the 2011–2012 survey years. We added the means of MiBP and MnBP as an estimate of the geometric mean concentration of MBP (27.7 µg/g), which was much lower than the results for both the ASD (51%) and TD (50%) groups from our study [48]. Using the same method for estimating the mean concentration of MBP, results from the NHANES pilot study on children 3–5 years for the concentration of MBP (40.0 µg/g) is more comparable to our results [57]. Additionally, the creatinine-adjusted geometric mean concentration of MEHHP in our study was about 30% higher for children with ASD when compared to TD children (14.94 µg/g vs. 11.08 µg/g). The geometric mean concentration of 14.9 µg/g as reported by NHANES (2011–2012) for children ages 6–11 years is comparable to our findings for children with ASD, but 26% higher than TD children [48]. Since the geometric mean MEHHP concentration shows a decreasing trend over NHANES survey years 2001–2002 to 2011–2012, our findings for TD children could possibly be comparable to the current population average, while our findings for ASD children may be elevated as compared to the current population average. In the NHANES pilot study on children 3–5 years, the geometric mean concentration of MEHHP (20.3 µg/g) was higher when compared to our results [57].

Creatinine-adjusted geometric mean concentrations of MEP, MEOHP, and MCPP from the children with ASD and the TD children in this study were comparable (<20% difference) with creatinine-adjusted geometric mean concentrations reported by NHANES for survey years 2011–2012 [48]. Results of the NHANES pilot study of children 3–5 years were higher than our results for MEP (42.6 µg/g) and MEOHP (13.2 µg/g), and were comparable to our results for MCPP (3.9 µg/g) [57]. According to NHANES data, the mean PEM concentrations in all of the age groups have a decreasing trend across all of the reported survey years [58].

Di-(2-ethylhexyl) phthalate (DEHP) has many metabolites, including MEHHP, MEOHP, and MEHP. While we were unable to report mean concentrations of MEHP due to a large proportion below LoD, Testa et al. reported that among Italian children, ASD cases had significantly higher urine creatinine-adjusted median MEHP concentrations when compared to controls [8]. Stein et al. further separated MEHP into subsequent metabolites and found that ASD cases had a significantly higher mean free MEHP as compared to controls after adjusting for sex, age, BMI, and creatinine. However, they also found that controls had significantly higher mean % bound MEHP when compared to ASD cases after adjusting for the same potential confounders [59]. Kardas et al. also reported higher mean serum MEHP concentration among ASD cases as compared to controls [11]. 

### 4.4. Routes of Exposure to PEMs

Phthalates are widely used in manufacturing plastic products but are not covalently bound to them, allowing them to shed and to become ubiquitous in the environment worldwide [60]. Therefore, there are multiple routes of human exposure to phthalates, including plastic containers, household dust [37], water, dirt, and food products such as meat, dairy, fruits, soft drinks, and fish [38]. Studies have also shown that exposure to phthalates that occur during pregnancy [61] and breastfeeding [62] may have an adverse effect on fetal and infant health. A study conducted in the UK concluded that DEHP and dibutyl phthalate (DBP) (and their respective metabolites, MEHP and MnBP) were found in several animal based food products. DEHP was found in the highest concentrations, and the main route of exposure to young children was through the consumption of fish, meat, and milk [39]. DEHP has been reported to have the highest production volume [60]. In our study, MEHP had about 70% of results below the limit of detection. However, we found that creatinine-adjusted concentrations of MEOHP, another metabolite of DEHP, were significantly lower among those who drank juices, such as orange juice and tomato juice, when compared to those who did not drink juice.

Phthalate levels in household dust were found to have no association with ASD, however DEHP and Benzylbutylphthalate (BBzP) concentrations in dust were associated with an increased risk of Developmental Disability (DD) and impairments in communication and daily living skills among TD children. Additionally, higher concentrations of DBP and DEP in dust were associated with hyperactivity in boys with ASD or DD [37]. We did not find any statistically significant associations between DEHP and BBzP metabolites or any other PEMs and ASD status of children, however, our study was underpowered due to limited sample size. The association between urine PEM concentrations and ASD status requires further investigation.

## 5. Limitations

This was a pilot study intended to assess the feasibility of conducting a case-control study of ASD, and we acknowledge certain limitations. Although the original study was designed as a 1:1 matched case-control, we encountered difficulty enrolling TD children. We believe that among other factors, this difficulty can largely be attributed to the reluctance of parents to have their TD children participate in a blood draw that would not directly benefit their child. Although several strategies were employed to enroll controls, including advertising in local papers and working with local pediatricians, we only successfully enrolled 10 TD children. This potentially contributed to selection bias in the controls and decreased the generalizability of our findings. Additionally, due to the limited sample size, we may not have adequate power for testing the statistical significance of differences between comparison groups. As a result of this limitation, we did not adjust for potential confounding variables, such as maternal and paternal age or education, when assessing the associations between analyte concentrations and ASD status to avoid reporting unstable estimates from the regression models. We also acknowledge the potential correlation between ASD cases and TD controls for the nine matched pairs, who were matched on age, race, and gender, and modeling techniques that account for within-pair correlation, such as Generalized Estimating Equations or Mixed Effect Models, may have been more appropriate for the analysis of some analytes. However, due to the limited sample size, we decided to use GLM. Future studies that enroll a larger number of matched ASD cases and TD controls should account for such correlation. Furthermore, we acknowledge that we did not have data on the source of canned and packaged foods. Finally, we did not adjust for multiple comparisons, so it is possible that any statistically significant findings reported in this paper may be due to chance alone.

## 6. Conclusions

We reported lipid- or creatinine-adjusted mean concentrations of dioxins, dibenzofurans, BPA, and PEMs, in children 2–8 years of age, with and without ASD along the coast of the Gulf of Mexico near Alabama, which could serve as a reference for future studies. We did not find any significant differences in the adjusted mean concentrations of OCDD, BPA, or PEMs between ASD cases and TD controls. However, these findings are inconclusive and should be interpreted with caution due to the limited sample size. We also compared the concentrations of chemicals that were analyzed in this study with those reported by NHANES. Furthermore, we found that certain dietary exposures, such as fish consumption, may be associated with higher lipid-adjusted mean OCDD concentrations. In addition, we found that dietary exposures to soft drinks and juices may be associated with lower creatinine-adjusted mean BPA and MEOHP concentrations, respectively. Future studies are required to replicate these findings.

## Figures and Tables

**Table 1:** Characteristics of children and their parents by autism spectrum disorder (ASD) case status (based on 30 ASD cases and 10 typically developing (TD) controls).

Variables	Categories	ASD Case (*n* = 30) N (%)	TD Control (*n* = 10) N (%)	*p*-Values
**Child’s sex**	Male	25 (83.3)	7 (70.0)	0.37
**Child’s age (months)**	Age < 72	16 (53.3)	5 (50.0)	0.85
	Age ≥ 72	14 (46.7)	5 (50.0)	
**Child’s race**	African American	13 (43.3)	3 (30.0)	0.46
	White	17 (56.7)	7 (70.0)	
**Maternal age** **(at child’s birth) ^a^**	<35 years	21 (72.4)	9 (90.0)	0.28
	≥35 years	8 (27.6)	1 (10.0)	
**Paternal age** **(at child’s birth) ^b^**	<35 years	15 (55.6)	7 (70.0)	0.43
	≥35 years	12 (44.4)	3 (30.0)	
**Maternal race ^a^**	African American	13 (44.8)	3 (30.0)	0.41
	White	16 (55.2)	7 (70.0)	
**Paternal race ^c^**	African American	13 (46.4)	3 (30.0)	0.37
	White	15 (53.6)	7 (70.0)	
**Maternal education** **(at child’s birth) ^a^**	Up to high school ^†^	14 (48.3)	3 (30.0)	0.32
	Beyond high school ^††^	15 (51.7)	7 (70.0)	
**Paternal education** **(at child’s birth) ^d^**	Up to high school ^†^	11 (40.7)	4 (50.0)	0.64
	Beyond high school ^††^	16 (59.3)	4 (50.0)	
**Socioeconomic status (SES) ^c^**	Home ownership	14 (50.0)	7 (70.0)	0.28

**^a^** Data were missing for 1 ASD case, ^b^ Data were missing for 3 ASD cases, ^c^ Data were missing for 2 ASD cases, ^d^ Data were missing for 3 ASD cases and 2 TD controls, ^†^ Up to high school education means attended Primary/Jr. Secondary, and Secondary/High/Technical schools, ^††^ Beyond high school education means attended a Vocational, Tertiary College, or University.

**Table 2:** Limit of detection (LoD) of dioxin and dibenzofuran analytes in serum (N = 40) as compared with LoDs reported by the National Health and Nutrition Health Examination Survey (NHANES).

Exposure Category	Analyte	LoD Range (pg/g)	Below LoD (N (%))	NHANES Mean (pg/g) *	NHANES LoD (pg/g) *
**Dioxins**	2,3,7,8-TCDD	3.36–12.4	40 (100.0)	<LoD	3.8
	1,2,3,7,8-PECDD	3.36–12.4	37 (92.5)	<LoD	4.5
	1,2,3,4,7,8-HXCDD	8.41–31.0	40 (100.0)	<LoD	11.9
	1,2,3,6,7,8-HXCDD	8.41–31.0	37 (92.5)	<LoD	12.3
	1,2,3,7,8,9-HXCDD	8.41–31.0	40 (100.0)	<LoD	12.3
	1,2,3,4,6,7,8-HPCDD	8.41–31.0	24 (60.0)	16.7	13.0
	OCDD	8.41–31.0	0 (0.0)	<LoD	218.0
	Total tetra-dioxins	3.36–12.4	40 (100.0)	NR	NR
	Total penta-dioxins	3.36–12.4	37 (92.5)	NR	NR
	Total hexa-dioxins	8.41–31.0	38 (95.0)	NR	NR
	Total hepta-dioxins	8.41–31.0	29 (72.5)	NR	NR
**Dibenzofurans**	2,3,7,8-TCDF	3.36–12.4	40 (100.0)	<LoD	6.0
	1,2,3,7,8-PECDF	3.36–12.4	40 (100.0)	<LoD	7.1
	2,3,4,7,8-PECDF	3.36–12.4	30 (75.0)	<LoD	6.8
	1,2,3,4,7,8-HXCDF	8.41–31.0	40 (100.0)	<LoD	7.4
	1,2,3,6,7,8-HXCDF	8.41–31.0	40 (100.0)	<LoD	7.9
	1,2,3,7,8,9-HXCDF	8.41–31.0	40 (100.0)	<LoD	8.3
	2,3,4,6,7,8-HXCDF	8.41–31.0	40 (100.0)	<LoD	8.2
	1,2,3,4,6,7,8-HPCDF	8.41–31.0	38 (95.0)	9.36	8.6
	1,2,3,4,7,8,9-HPCDF	8.41–31.0	40 (100.0)	<LoD	8.6
	OCDF	8.41–31.0	39 (97.5)	<LoD	12.0
	Total tetra-furans	3.36–12.4	40 (100.0)	NR	NR
	Total penta-furans	3.36–12.4	30 (75.0)	NR	NR
	Total hexa-furans	8.41–31.0	40 (100.0)	NR	NR
	Total hepta-furans	8.41–31.0	38 (95.0)	NR	NR

* NHANES survey years 2003–2004 (children ages 12–19 years) [48]; NR = Data are not reported.

**Table 3:** Geometric mean and standard deviation (SD) of BPA and mono phthalate esters concentrations in urine adjusted for creatinine (µg/g) by ASD case status (based on 24 ASD cases and 8 TD controls) compared with concentrations reported by National Health and Nutrition Examination Survey (NHANES).

Exposure Category	Analyte	LoD Range (ng/mL)	Below LoD N (%)	Adjusted for Creatinine (µg/g)					NHANES ^†^ Adjusted for Creatinine (µg/g)	
				ASD Case		TD Control		*p*-Value **		
				Mean * (SD)	Range	Mean * (SD)	Range		Mean	LoD *
**BPA**	Bisphenol A	0.25–0.34	6 (18.2)	1.33 (2.10)	0.26–6.72	0.93 (2.43)	0.16–3.13	0.27	2.27 **	0.4
**Mono Phthalate Esters**	Monomethyl phthalate (MMP)	0.98–8.61	26 (78.8)	NR	0.64–26.50	NR	0.26–18.89	NR	3.31	0.5
	Monoethyl phthalate (MEP)	0.98–4.08	3 (9.1)	28.93 (1.86)	7.77–84.00	37.61 (7.58)	1.28–2108.87	0.57	33.4	0.6
	Monobutyl phthalate (MBP) [sum of mono-*n*-butyl (MnBP) and mono-iso-butyl phthalate (MiBP)]	0.98–3.86	0 (0.0)	41.82 (1.88)	12.33–308.73	41.49 (2.60)	19.48–334.59	0.98	27.7 (15.9 + 11.8) ^††^	0.4, 0.2 ^††^
	Monobenzyl phthalate (MBzP)	0.98–2.08	1 (3.0)	19.01 (2.38)	3.20–96.83	12.55 (3.07)	1.51–70.58	0.28	12.5	0.3
	Mono-2-ethylhexyl phthalate (MEHP) (DEHP Metabolite)	0.98–1.03	23 (69.7)	NR	0.43–21.39	NR	0.55–5.54	NR	2.02	0.5
	Mono-(2-ethyl-5-oxohexyl) phthalate (DEHP Metabolite) (MEOHP)	0.99–4.59	0 (0.0)	9.76 (2.52)	1.93–117.99	8.23 (2.13)	3.93–39.26	0.64	9.93	0.2
	Mono-(2-ethyl-5-hydroxyhexyl) phthalate (DEHP Metabolite) (MEHHP)	0.98–3.96	2 (6.1)	14.94 (2.45)	2.25–144.36	11.08 (3.22)	0.91–46.86	0.46	14.9	0.2
	Mono-(3-carboxypropyl) phthalate (MCPP)	1.02–2.83	5 (15.2)	4.04 (1.74)	1.74–10.63	4.49 (1.75)	2.16–9.60	0.64	4.79	0.2
	Mono-cyclohexyl phthalate (MCHP)	0.98–1.57	32 (97.0)	NR	0.43–7.77	NR	0.26–1.28	NR	<LoD	0.4
	Mono-iso-nonyl Phthalate (MiNP)	0.98–1.16	33 (100.0)	NR	NR	NR	NR	NR	<LoD	0.6

NR = Data are not reported because over 69% of samples were below their respective LoD; * Mean indicates the geometric mean = Exp. [Mean (ln of creatinine-adjusted chemical concentration)]; ** *p*-value from GLM or linear regression model; ^†^ NHANES survey years 2011–2012 (children ages 6–11 years) [48]. ^††^ NHANES did not directly report data for MBP, so we have estimated the mean of MBP by adding mean MnBP and MiBP concentrations and we listed both LoDs, respectively.

**Table 4:** Associations between dietary consumption and lipid-adjusted octa-chlorinated dioxin (OCDD) (pg/g) concentrations in serum (N = 39) ^a^.

Exposure Variables	Category	Yes		No		*p*-Value ^b,c^
		Mean * (SD)	N	Mean * (SD)	N	
**Imported Seafood Consumption**	Ate salt water fish	79.26 (1.67)	8	84.34 (2.18)	31	0.79
	Ate fresh water fish (salmon, tilapia, catfish)	113.81 (2.43) ^†^	11	73.65 (1.88)	28	0.11
	Ate sardine, mackerel (canned fish)	263.75 (9.75) ^†^	2	78.24 (1.79)	37	0.02
	Ate tuna (canned fish)	123.33 (2.88) ^†^	8	75.24 (1.82)	31	0.10
	Ate shrimp	115.03 (2.46) ^†^	11	73.34 (1.86)	28	0.09
	Ate shellfish (lobster, crab)	95.04 (2.06)	3	82.36 (2.09)	36	0.83
**Fresh Seafood Consumption**	Ate farm-raised tilapia or catfish	107.13 (3.13) ^†^	7	78.80 (1.85)	32	0.33
	Ate lake/pond fish (catfish, crappie)	59.14 (1.43)	3	85.68 (2.11)	36	0.45
	Ate bay fish (speckled trout, redfish, flounder)	87.48 (1.69)	5	82.67 (2.14)	34	0.92
	Ate river fish (bass, trout)	74.68 (3.01)	3	84.03 (2.03)	36	0.85
	Ate offshore fish (tuna, snapper, whiting)	74.02 (1.31)	5	84.72 (2.17)	34	0.66
	Ate shellfish (lobster, crab, crawfish)	124.58 (2.66) ^†^	12	69.62 (1.66)	27	0.02
	Ate mussels (clams, oysters, scallops)	153.21 (6.46) ^†^	3	79.14 (1.79)	36	0.14
**Meat/organ Consumption (as main dish)**	Beef	87.17 (2.15)	30	71.48 (1.84)	9	0.53
	Pork	89.14 (2.13)	30	66.36 (1.83)	9	0.31
	Animal fat (used for cooking)	105.43 (2.87)	10	76.76 (1.78)	29	0.20
**Dairy Product and Egg Consumption**	Milk	76.07 (1.81)	35	183.76 (3.78) ^†^	4	0.01
	Cheese	81.32 (1.98)	33	94.84 (2.76)	6	0.56
	Yogurt	84.51 (2.10)	29	79.78 (2.07)	10	0.86
	Eggs	83.73 (2.09)	28	82.11 (2.10)	11	0.98

* Mean indicates the geometric mean = Exp. [Mean (ln of lipid-adjusted OCDD)]; The “Yes” column includes participants who met the category specified in front of each exposure variable; The “No” column includes participants who did not meet the category specified in front of each exposure variable; ^a^ Food frequency data are missing for 1 child; ^b^ *p*-value from GLM; ^c^ Adjusted for case status (ASD case or TD control); ^†^ Extreme observation is present but the value has been verified to be true, hence kept in analysis.

**Table 5:** Associations between dietary consumption and creatinine-adjusted dibenzofurans, bisphenol A (BPA) (µg/g) concentrations in urine (N = 31) ^a^.

Exposure Variables	Category	Yes		No		*p*-Value ^b,c^
		Mean * (SD)	N	Mean* (SD)	N	
**Bottled water used for drinking**		1.07 (2.30)	13	1.21 (1.95)	18	0.64
**Canned fish consumption**	Tuna	1.79 (1.83)	5	1.05 (2.07)	26	0.10
**Juices/soft drinks**	Juices (e.g., orange, tomato, etc.)	1.22 (2.06)	25	0.90 (2.15)	6	0.42
	Flavored beverages	1.18 (2.12)	26	1.01 (1.91)	5	0.50
	Soft drinks (Soda, Coke/Pepsi)	0.82 (1.95)	19	1.96 (1.59)	12	<0.01
	Hot tea (e.g., Black, Earl Grey, Green)	1.10 (1.79)	4	1.16 (2.13)	27	0.95
	Iced Tea	1.14 (1.91)	14	1.16 (2.25)	17	0.88
**Canned food (vegetables, soups, seafood, etc.)**		1.12 (2.27)	23	1.23 (1.49)	8	0.89

* Mean indicates the geometric mean = Exp. [Mean (ln of creatinine-adjusted BPA)]; The “Yes” column includes participants who met the category specified in front of each exposure variable; The “No” column includes participants who did not meet the category specified in front of each exposure variable; ^a^ Food frequency data were missing for 1 child; ^b^ *p*-value from GLM; ^c^ Adjusted for case status (ASD case or TD control).

**Table 6:** Associations between dietary consumption and creatinine-adjusted Mono-(2-ethyl-5-oxohexyl) phthalate (MEOHP) (µg/g) concentrations in urine (N = 31) ^a^.

Exposure Variables	Category	Yes		No		*p*-Value ^b,c^
		Mean * (SD)	N	Mean * (SD)	N	
**Bottled water used for drinking**		7.36 (2.01)	13	11.68 (2.60)	18	0.15
**Canned fish consumption**	Tuna	9.32 (1.31)	5	9.68 (2.59)	26	0.99
**Juices/soft drinks**	Juices (e.g., orange, tomato, etc.)	8.31 (2.06)	25	17.79 (3.45)	6	0.05
	Flavored beverages	9.48 (2.02)	26	10.41 (5.09)	5	0.95
	Soft drinks (Soda, Coke/Pepsi)	8.78 (2.02)	19	11.12 (3.05)	12	0.53
	Hot tea (e.g., Black, Earl Grey, Green)	8.75 (2.31)	4	9.76 (2.45)	27	0.92
	Iced Tea	10.89 (2.02)	14	8.70 (2.74)	17	0.52
**Canned food (vegetables, soups, seafood, etc.)**		9.75 (2.04)	23	9.28 (3.67)	8	0.81

* Mean of chemical indicates the geometric mean = Exp. [Mean (ln of creatinine-adjusted MEOHP)]; The “Yes” column includes participants who met the category specified in front of each exposure variable; The “No” column includes participants who did not meet the category specified in front of each exposure variable; ^a^ Food frequency data are missing for 1 child; ^b^ *p*-value from GLM; ^c^ Adjusted for case status (ASD case or TD control).
